# The COVID-19 Psychological Wellbeing Study: Understanding the Longitudinal Psychosocial Impact of the COVID-19 Pandemic in the UK; a Methodological Overview Paper

**DOI:** 10.1007/s10862-020-09841-4

**Published:** 2020-11-04

**Authors:** Cherie Armour, Emily McGlinchey, Sarah Butter, Kareena McAloney-Kocaman, Kerri E. McPherson

**Affiliations:** 1 Stress Trauma and Related Conditions (STARC) Research Lab, School of Psychology, Queen’s University Belfast, Belfast, Northern Ireland UK; 2 Department of Psychology, Glasgow Caledonian University, Glasgow, Scotland UK

**Keywords:** Psychosocial, COVID-19, Coronavirus, Mental health, General population, Longitudinal, Survey, United Kingdom

## Abstract

The COVID-19 Psychological Wellbeing Study was designed and implemented as a rapid survey of the psychosocial impacts of the novel severe acute respiratory syndrome coronavirus 2 (SARS-CoV-2), known as COVID-19 in residents across the United Kingdom. This study utilised a longitudinal design to collect online survey based data. The aim of this paper was to describe (1) the rationale behind the study and the corresponding selection of constructs to be assessed; (2) the study design and methodology; (3) the resultant sociodemographic characteristics of the full sample; (4) how the baseline survey data compares to the UK adult population (using data from the Census) on a variety of sociodemographic variables; (5) the ongoing efforts for weekly and monthly longitudinal assessments of the baseline cohort; and (6) outline future research directions. We believe the study is in a unique position to make a significant contribution to the growing body of literature to help understand the psychological impact of this pandemic and inform future clinical and research directions that the UK will implement in response to COVID-19.

## Introduction

The psychosocial effects of the severe acute respiratory syndrome coronavirus 2 (SARS-CoV-2), otherwise known as COVID-19 are pervasive and of significant societal concern. Indeed, it is likely that COVID-19 will not only affect the mental health of the population presently, as the pandemic spreads, but that the impact may last long into the future. We would expect that many individuals will experience a rise in mental distress symptoms, such as anxiety and depression, during these unprecedented times when populations have been required to drastically change their day to day way of life. However, there is further concern that for some, particularly those with pre-existing vulnerabilities, this rise in mental distress will reach clinically significant levels and in turn affect day to day functioning. This is expected due to the rapidly changing and uncertain situation that is COVID-19 and the very real fears that people will have for themselves and others around infection and mortality. Previous research on SARS, MERS and H1N1 (Swine) flu, have given insight into the psychological impact of the outbreak of an infectious respiratory disease and the measures taken to curb its spread. A high degree of psychological distress was reported during such pandemics, particularly among healthcare workers, quarantined individuals, and SARS survivors and their family members (Brooks et al. 2020; Gardner and Moallef 2015; Maunder 2004; Tsang et al. 2004). Moreover, recent research from the initial phases of the COVID-19 outbreak in China has suggested that there has been a significant psychological impact on the general population (Qiu et al. 2020; Wang et al. 2020).

In response to the COVID-19 pandemic, the UK governments put in place several restrictions as the situation progressed. On January 31^st^ 2020, the first coronavirus case was confirmed in the UK (exactly 1 month after the first COVID-19 case was detected in Wuhan, China). On March 11^th^, WHO declared COVID-19 a pandemic. On March 19^th^ The Coronavirus Bill 2019–21 was introduced in the House of Commons. On March 23^rd^, the UK Prime Minister Boris Johnston announced severe restrictions (enforceable by police), including a need for the population to stay at home unless there was an absolute necessity to leave such as shopping for food, medical emergencies, or if required to work in a government designated keyworker role (a comprehensive timeline from 31.12.2019 – 23.03.2020 can be found in McBride et al. 2020; pre-print).

On April 16^th^ the UK lockdown was extended for “at least” another 3 weeks. However, by this time both Wales (April 8^th^), NI (April 15^th^) and Scotland (afternoon of April 16^th^) had separately announced extensions to the lockdown beyond the initial three-week period. By May 5^th^, the UK’s death toll was the highest in Europe and the second highest in the world. On May 10^th^ the UK Prime Minister announced an initial easing of lockdown restrictions. In this address, those in England who could not work from home were “actively encouraged” to return to work (avoiding public transport if possible and if not possible to social distance and wear face coverings), an unlimited amount of outdoor exercise was allowed, and individuals could drive to outdoor destinations. However, the administrations with Scotland, Wales and NI chose not to adopt the ‘Stay Alert’ strategy brought forward by the UK Prime Minister and set out their own plans on easing lockdown restrictions. From May 11^th^ both the Scottish and Welsh Governments eased restrictions to allow more than once daily exercise. No other changes were made to the lockdown restrictions in Scotland; however, Wales began opening gardening and recycling centres. The NI Executive agreed a similar three-week extension and permitted garden and recycling centres to reopen (May 18^th^). Furthermore, NI also began recommending use of face masks in enclosed spaces (May 8^th^), a measure announced in Scotland weeks earlier (April 28^th^). While there are plans in England to begin opening schools by June 1^st^, Wales, Scotland and NI have all indicated that schools will likely not open until the new academic year. Additionally, a phased strategy to ease lockdown restrictions was outlined by the Prime Minister on the May 11^th^. However, the implementation of each of the phases involved in easing the population out of lockdown and the opening of various business and places is subject to continuous review based on the most up to date information regarding the virus. It is important to mention each nation within the UK differs regarding their individual phased strategy regarding the removal of lockdown restrictions.

At the initiation of lockdown (March 23^rd^ 2020), 359 COVID-19 deaths^1^ had been officially reported across the UK (England: 331, Wales: 16, Scotland: 10 and NI: 2). By the time the COVID-19 Psychological Wellbeing baseline survey closed (April 24^th^), official figures stated that 22,792 COVID-19 deaths had taken place across the UK (England: 20,658, Wales: 751, Scotland: 1,120 and NI: 263). At the time of writing this manuscript (May 25^th^) there have been 36,793 COVID-19 deaths in the UK (England: 32,760, Wales: 1,267, Scotland: 2,261 and NI: 505). Moreover, as of May 25^th^, when adjusting for population, the UK had the highest rate of daily confirmed COVID19 deaths worldwide (7 day average), approximately 5 people per million per day (Our World in Data 2020).

The government restrictions, coupled with an already increasing prevalence of mental ill health in the UK (Ford and McManus 2020; McManus et al. 2014), and a known adverse psychological impact of restrictions such as self-isolation; which intensify loneliness and reduce sense of connectedness, purpose and meaning in personal lives, has the potential to accelerate the prevalence rates of mental ill health across the UK. In recognising this, a team of leading mental health scientists published a position paper detailing a number of mental health research priorities for the UK in response to the COVID-19 pandemic. These included the need for increased monitoring and reporting of the rates of mental health issues and a need to determine the factors that adversely or positively affect mental health during this time. From a public health perspective, these priorities focused on the general population as well as specific populations (Holmes et al. 2020).

In line with the research priorities highlighted by Holmes et al. (2020), the COVID-19 Psychological Wellbeing Study assessed commonly occurring mental health disorders such as anxiety and depression among all participants. Furthermore, following the traumatic impact of previous outbreaks (Maunder 2004; Maunder et al. 2004; Wu et al. 2005), posttraumatic stress symptoms were measured, and, given the nature of the study and prior research having highlighted that quarantined and infected individuals and their family members were at increased risk of poor mental health (Brooks et al. 2020; Gardner and Moallef 2015; Tsang et al. 2004), we also queried specific COVID-19 exposure experiences.

Separate from mental health difficulties, a range of more practical concerns related specifically to the pandemic, such as adherence with government advice, concerns about school closures, governments perceived efficiency, job security, financial implications, the capacity of the health service, and infection concern were examined. Such concerns, while distressing themselves, may also contribute to a deterioration in mental health and wellbeing. Individuals who are concerned about becoming infected or about the availability of healthcare may be at risk of developing health-related anxiety or obsessive health behaviours (Abba-Aji et al. 2020; Asmundson and Taylor 2020; Blakey and Abramowitz 2017; Jungmann and Witthöft 2020). Individuals suffering from a job loss or financial instability as a result of the pandemic may be at greater risk of developing a range of mental health issues (Mental Health Foundation 2020). Moreover, the substantial changes to daily life as a result of lockdown restrictions has added stress to many individuals’ work and family lives (e.g. school closures forcing parents to home-school their children while working from home themselves). Such disruption to normal routine, activities and livelihoods may lead to increases in depression, loneliness, self-harming and suicidal behaviour, and harmful alcohol and drug use (WHO 2020). Finally, the role of media consumption in amplifying distress was included; this was subsequently highlighted as a mental health research priority (Holmes et al. 2020).

As previously mentioned, it is important to identify groups of individuals who are most at risk of poor mental health during this time in order to help guide experts and to formulate an appropriate, proportionate response to these needs. Indeed, older individuals and those with physical health problems may be at risk of more severe outcomes if infected with coronavirus and thus may also experience greater levels of concern and distress (Holmes et al. 2020; Shevlin et al 2020; preprint). Individuals with prior and current mental ill health concerns may have exacerbated distress due to disruption in services and increased isolation (Elovainio et al. 2017; Holmes et al. 2020), and individuals with lower incomes or financial instability may have less access to technology (Hernandez and Roberts 2018) and more difficult housing situations (Eurostat 2019). The COVID-19 Psychological Wellbeing Study therefore investigated a range of sociodemographic characteristics to help understand which groups were potentially the most psychologically impacted.

The primary aim of this paper is to report the study protocol and the resultant sociodemographic characteristics of the participants of the COVID-19 Psychological Wellbeing baseline survey. For completeness, although never the intention, the sample proportions will be mapped to the UK adult population proportions (using data from the Census). This will allow readers to determine areas where the sample data approximates and thus represents the UK general population and areas where particular sociodemographic may be over- and/or under-represented. Our secondary aim was to provide a clear and concise account of the data that has been collected across the UK population and sub-divided by UK nation (England/Wales, Scotland, and Northern Ireland). We aim to conclude with a summary of the ongoing efforts for weekly and monthly longitudinal assessments of the baseline cohort.

## Method

### Study Design

The COVID-19 Psychological Wellbeing Study is a longitudinal, multi-wave online survey of the adult (18 years +) general population of the UK. The study was designed to rapidly assess and monitor the psychosocial impact of the COVID-19 pandemic on UK residents. This was achieved by implementing an online survey; launched on March 23^rd^ 2020 and closed on April 24^th^ 2020. Participants who completed the initial survey were asked to complete follow up surveys on a weekly basis for three weeks (from their completion of the baseline survey [Baseline as T1 + T2 = week 1, T3 = week 2, T4 = week3]) and then at three monthly intervals post baseline survey completion [Baseline as T1 + T2 = month 1, T3 = month 2, T4 = month 3]). This study design result in four waves of weekly longitudinal data during the first month of the UK lockdown and four waves of longitudinal data during the 4-month period from the 1^st^ day of the UK lockdown. The former allows us to track mental health outcomes during an intense period of lockdown when restrictions were at their height and the latter allows us to track mental health outcomes over a longer period in which lock down restrictions are eased.

### Participants

Participants were recruited via two avenues (1) a large-scale social media campaign and (2) using an online participant panel called Prolific. All participants were required to be 18 + years or older, currently resident in the UK and able to read and write in English. No other exclusion criteria were applied. Participation was voluntary. Those who participated via social media recruitment activities were included into a prize draw for one of six £150 vouchers. Participants who joined the study via Prolific received between £1.00 and £2.00 depending on survey length across baseline and follow-ups.

### Procedures

Data collection commenced on March 23^rd^ 2020. This timeline corresponds with the commencement of the UK’s period of lockdown whereby the UK Prime Minister announced that all people were required to stay at home except for specific and essential reasons to leave (March 23^rd^; see timeline above). The survey was administered entirely online through the survey data collection platform ‘Qualtrics’. The study was launched initially via a variety of social media platforms (Twitter, Facebook). Additional data was collected using a panel of UK residents hosted by Prolific (https://www.prolific.co/).

All participants, regardless of recruitment mode were required to complete the baseline survey. All those recruited via social media were asked to complete follow up surveys on a weekly basis for three weeks and then at monthly intervals (1 month, 2 months and 3 months). Participants recruited via Prolific were asked to complete the monthly follow up surveys only due to a lack of financial resources that would have been required for such extensive weekly surveys.

As surveys progressed, certain topics were added to the survey battery to answer key political and scientific topics of interest and therefore there are additions to measures and topics assessed across weeks and months. For the purpose of this paper, we focus on all measures included in the baseline data as completed by all participants.

### Informed Consent

All participants received a detailed participant information sheet outlining the purpose of the study, exact details concerning participation, how information would be stored, what would happen to the information concerning onward publication of the data and the results, and the risks and benefits associated with participation. Participants were informed about confidentiality and under what circumstances confidentiality would be broken. Likewise, they were informed that participation was voluntary and they were free to refuse to participate at any point and therefore free to refuse to complete further surveys. Participants were informed that if they wished to withdraw they had to contact the Chief Investigator with their email address and mobile telephone number used for the study and request that no more reminders about participation were sent to them. They were informed that the decision to withdraw would bring no negative consequences to them nor affect their relationship with the researchers, support providers, or Queen’s University. They were additionally provided with details on the formal complaints procedure, contact details for the researchers, ethical approval, and where they could access the most up-to-date information concerning COVID-19. Subsequently, participants were presented with 10 statements, which they had to confirm in order to provide full consent to participate in the study.

### General Data Protection Regulation (GDPR) Compliance

All study procedures were in accordance with GDPR. Personal contact data was separated from the survey responses and replaced with a unique identifier. Personal contact data is stored in a separate database and access is restricted to two members of the research team. All data will be fully anonymised prior to being shared across researchers in the team.

### Ethical Approval

Ethical approval for the COVID-19 Psychological Wellbeing study was provided by the ethical review panel in the faculty of Engineering and Physical Sciences at Queen’s University Belfast (Reference: EPS 20_96) and also Glasgow Caledonian University Health and Life Sciences Ethics Committee, (HLS/PSWAHS/19/157). We are grateful to Professor Brendan Murtagh (the Chair) and additional reviewers for their rapid review and approval of this study.

### Quality Control

A number of quality control measures were applied to the survey to help ensure the authenticity of responses and screen out those did not meet the inclusion criteria. The survey was firstly piloted by the research team as a measure of quality control (*n* = 10) before going live on social media and Prolific.

Individuals were removed from the data if;

(i) The respondent clicked into the survey link but did not complete any measures (*n* = 205),
(ii) The respondent did not provide full informed consent (*n* = 27),
(iii) The respondent did not provide information relating to the inclusion criteria (i.e. age and/or current residency; *n* = 113),
(iv) The respondent did not meet the inclusion criteria (i.e. < 18 years or non-UK resident, *n* = 107),
(v) The respondent completed the survey in less than the minimum completion time (*n* = 60). Minimum completion time was set at 483 s (8 min, 3 s), half of the median completion time for the sample.
(vi) Data were screened for uniformity of responses; however, no responses were removed based on this criterion.
(vii) This resulted in 1989 eligible responses.

## Measures

A series of standardised self-report measures were included in the survey. Many were included in full, whereas some were included in part. The survey also included newly created questions pertaining to COVID-19 exposures, worries, and symptoms. Furthermore, we included a series of questions related to social and traditional media engagements around COVID-19 news. This study was devised in early March and therefore there were no standardised measures available covering the COVID-19 pandemic. To ensure our questions were robust and reliable several academics reviewed them in full and suggested modifications based on the extant knowledge of COVID-19 at the time. This knowledge was based on reliable and trusted sources such as Public Health England, the National Health Service, and the World Health Organisation. These modifications were incorporated in full for the final survey. Further details are below:

### Sociodemographic Variables

Participants provided information regarding their gender, age, marital status, ethnicity, religious status, personal income, and their highest level of educational attainment. Female participants were asked to specify if they were currently pregnant. Participants were also asked to provide information related to education and employment and to indicate whether they themselves or their family members were working in one of the government assigned key worker roles. If they indicated that a family member was a key worker, they were also asked to indicate if they lived as part of the same household.

Participants were asked several questions based on their residential status. Specifically, their place of residence, residence type (*‘house’, ‘room in a shared house’, ‘apartment/flat’, ‘student halls’, ‘residential home’ or ‘other*), urban vs rural (‘*isolated dwelling’, ‘hamlet’, ‘village’, ‘small town’, ‘large town’, ‘city’*), and number of bedrooms in place of residence. They were also asked to best describe their housing situation (*‘owned outright’, ‘owned with mortgage’, shared ownership’, rented’, ‘living rent free’ or ‘other’*). Finally, participants were also asked to specify the number of adults over 18 years and children under 18 years present in their place of residence, and whether they currently owned any pets (and were asked to specify what type or types of pets they had).

### Previous Physical or Mental Health Conditions

The survey queried whether participants had ever suffered from a physical or mental health related concern. Specifically, asthma, heart disease, cancer, diabetes, shortness of breath, post-traumatic stress disorder, major depressive disorder, phobia, social phobia, obsessive compulsive disorder, generalised anxiety disorder, psychotic disorders, eating disorders, health anxiety or another kind of chronic condition not specified.

### COVID-19

#### COVID-19 Living Status

Participants were asked to indicate their current living status in relation to COVID-19 at the time of completing the baseline survey (*‘I am living as normal’, ‘I am not self-isolating but have cut down my usual activities as a precaution’, ‘I am not self-isolating but have been told to work from home’, ‘I am self-isolating as I do not want to get ill, but I am not high risk, ‘I am self-isolating as I do not want to get ill, but I am regarded as high risk’, ‘I am self-isolating as I do not want others to get ill’, ‘I have been told to self-isolate due to possible symptoms of COVID-19′, ‘I have been told to self-isolate due to a diagnosis of COVID-19′, or ‘I have been ordered by the government or local authority to self-isolate/stay home’.*

#### COVID-19 Related Experiences

Participants were presented with a series of questions in relation to symptom expression, testing, diagnosis (for themselves or loved ones) and exposure to COVID-19 related deaths. Specifically, they were asked (at the time of survey completion) did they know someone who currently has or had in the past been quarantined for COVID-19 due to exposure and whether any of these people have been close family members or friends. Likewise, they were asked to indicate did they know someone who currently has or had in the past been diagnosed with COVID-19 and whether any of these people have been close family members or friends. Participants were also asked to indicate if they were a carer for someone who had been diagnosed with COVID-19.

Participants were also asked whether they themselves were currently in quarantine or had been in quarantine in the past due to COVID-19, whether they had self-isolated in order to avoid infection and whether they were regarded as ‘high risk’. Participants were also asked if they had self-isolated due to government advice and whether they had self-isolated because they had symptoms. Participants were also asked whether they themselves have been tested for COVID-19 and whether they had been diagnosed with COVID-19. Two questions pertained to whether participants had received a flu vaccination in the past year and whether they had had the flu in the past year. Participants were also asked about exposure to COVID-19 related deaths, specifically whether they had experienced the death of a close friend or family member and whether they had been exposed to COVID-19 related deaths due to their occupational role. Finally, participants were asked to indicate whether they experienced any of the following symptoms (*‘fever’, ‘cough’, ‘sore throat’, ‘headache’, ‘cold symptoms’ or ‘no symptoms’*) at the time of survey completion.

#### Media/Information Consumption

A number of questions queried participants’ media consumption in related to COVID-19. Specifically, they were asked how often they were watching, reading, and hearing reports or updates about COVID-19 on social media, on traditional media and on a dedicated app that has been set up to provide COVID-19 updates. The possible response categories in relation to each type of media consumption were, (1) *less than once a day*, (2) *1–5 times a day*, (3) *6–10 times a day*, (4) *11–20 times a day*, (5) *20–50 times a day*. and (6) *more than 50 times a day*.

#### COVID-19 Related Concerns

Participants were asked to indicate how worried they were about several COVID-19 related concerns. Specifically, worries around quarantine/self-isolation, being infected with the virus by others, infecting others with the virus, stigmatisation due to exposure, job security, the financial implications of the outbreak, food shortages, the government’s ability to manage the outbreak, the healthcare systems ability to care for COVID-19 patients, border closures and the impact of school/university closures on children and young adults. Each of these responses were rated on a Likert scale, ranging from 1 (*‘Not at all’*) to 5 (*‘Extremely’*). Finally, participants were asked to indicate if they thought school, university or border closures were necessary.

### Mental Health Variables

#### Trauma Exposure

Prior trauma exposure was assessed using the Life Events Checklist for DSM-5 (LEC-5; Weathers et al. 2013a). The LEC-5 contains 17 items measuring trauma exposure and therefore the measure is used to assess whether an individual has been exposed a PTSD ‘Criterion A’ traumatic event. In the current study we added an additional event of ‘Coronavirus’. Participants were asked if any of the 18 stressful life events, as measured by the LEC-5 (plus our single addition), ever happened to them. The possible response categories were *‘yes’* or *‘no’*. Participants were asked to keep their answers to the trauma screen in mind and indicate which event they felt was the worst. The possible options were, *‘Natural disaster’; ‘Fire or explosion’; ‘Transportation accident’; ‘Serious accident at work, home, or during recreational activity’; ‘Exposure to toxic substance’; ‘Physical assault’; ‘Assault with a weapon’; ‘Sexual assault,’; ‘Other unwanted or uncomfortable sexual experience’; ‘Combat or exposure to a war zone’; ‘Captivity’; ‘Life threatening illness (not COVID-19)’; ‘witnessing severe human suffering’; ‘Sudden violent death’; ‘Sudden accidental death’; ‘Serious injury, harm, or death you caused to someone else’; ‘Coronavirus’; ‘Other’; ‘None’*. Previous research has highlighted excellent psychometric properties of the LEC-5 (Gray et al. 2004).

#### COVID-19 Related Post-Traumatic Stress Disorder (PTSD)

PTSD was assessed with the PTSD Checklist for DSM-5 (PCL-5; Weathers et al. 2013b). The PTSD checklist contains 20 items reflect the DSM-5 symptom criteria for PTSD. In this study, participants were asked to think about their responses in regard to their COVID-19 related experiences. These 20 items are organised into one of four clusters each reflecting a different aspect of PTSD symptomatology. These clusters are *‘intrusions’*, *‘avoidance’*, ‘*negative alterations in cognition and mood’* and *‘alterations in arousal and reactivity’*. Each item of the PCL-5 is rated on a five-point Likert scale (‘*0* = *Not at all’* to *‘4* = *Extremely’*), and asks participants to indicate how much each symptom bothered them over the past month. A participant must rate a given item (or symptom) as *‘2* = *Moderately’* or higher in order to constitute as valid endorsement of a symptom. In order to meet the criteria for a diagnosis of PTSD, there must first of all be trauma exposure, followed by valid symptom endorsement across each of the PTSD symptom clusters. According to the DSM-5, this requires valid endorsement (a score of 2 or higher) of at least, one *‘intrusions’* item, one *‘avoidance’* item, two ‘*negative alterations in cognition and mood’* items and finally, two *‘alterations in arousal and reactivity’* items (American Psychiatric Association [APA] 2013). Additionally, previous empirical research suggests that a total score on the PCL-5 of between 34 is indicative of ‘probable PTSD’ (Murphy et al. 2017). In line with the research outlined above, if a respondent had a score of 34 or above on the PCL-5 they were classified as reporting ‘probable PTSD’. A wealth of previous literature has demonstrated the excellent psychometric properties of the PCL-5 across various populations (Blevins et al. 2015; Bovin et al. 2015; Weathers et al. 2013b; Wortmann et al. 2016).

#### Generalised Anxiety Disorder

The Generalised Anxiety Disorder scale (GAD-7; Spitzer et al. 2006) is a seven-item scale (GAD-7) used to measure symptoms of generalised anxiety disorder. The scores across all seven items are summed to yield a total score, with higher scores indicating higher levels of severity (range 0–21). The scale asks participants to reflect on the past two weeks in answering each of the seven items, with each item ranging from 0 (*not at all*) to 3 (*nearly every day*). Furthermore, across both adult and adolescent samples, scores on the GAD-7 have also been used to define severity of anxiety-based symptoms (Spitzer et al. 2006). A score of 0–4 is considered none/normal levels of anxiety, 5–9 is considered mild, 10–14 is moderate and 15–21 is severe (Spitzer et al. 2006). In adult samples scores of 10 or more may be of particular clinical concern, as they are likely to meet the diagnostic criteria for an anxiety disorder. Using the threshold score of 10, the GAD-7 has a sensitivity of 89% and a specificity of 82% for GAD (Spitzer et al. 2006). Therefore, in the context of this study scores of 10 or above were considered indicative of those meeting the criteria for GAD. Previous literature has demonstrated the excellent psychometric properties of the GAD-7 across various clinical and non-clinical populations (Kertz et al. 2013; Lee and Kim 2019; Rutter and Brown 2017; Spitzer et al. 2006).

#### Major Depressive Disorder

The Patient Health Questionnaire (PHQ-9; Kroenke et al. 2001), was used to measure symptoms of major depressive disorder. The PHQ-9 asks participants to reflect on the past two weeks in their response to nine items, which are based upon the DSM-IV diagnostic criteria (APA 2000) used to assess MDD symptomatology, namely, sleep, fatigue, concentration, low self-esteem, anhedonia, etc. However, it also is in line with the current DSM-5 criteria (APA 2013; Burdzovic and Brunborg 2017). Each item of the PHQ-9 is scored on a 4-point Likert scale, ranging from 0 to 3. The response categories were, *not at all* (0), *several days* (1), *more than half the days* (2) and *nearly every day* (3). Each item is summed to yield a total score, with a possible range of 0–27, with higher scores reflecting greater levels of MDD. Furthermore, scores on the PHQ-9 have also been used to define severity of MDD symptoms. In adults, a score of 0–4 none or mild, 5–9 is considered minimal, 10–14 is considered moderate, 15–19 is moderately severe, and ≥ 20 is severe. Furthermore, in adult samples scores of ≥ 10 or more may be of particular clinical concern, as they are likely to meet the diagnostic criteria for an MDD. Using the threshold score of ≥ 10, the PHQ-9 has a sensitivity of 88% and a specificity of 88% for MDD (Kroenke et al. 2001; Levis et al. 2019; Manea et al. 2012). Therefore, in the context of this study scores of 10 or above are considered as meeting the criteria for MDD. The PHQ-9 has been strongly supported for its applicability as a short screening tool (Burdzovic and Brunborg 2017) across various clinical and non-clinical contexts and support the psychometric validity of the scale (Allgaier et al. 2012; Burdzovic and Brunborg 2017; Lee et al. 2007; Levis et al. 2019; Titov et al. 2011; Umegaki and Todo 2017).

#### Sleep Quality

Participants were asked to rate what the quality of their sleep in general. The response categories were *‘very good’, ‘fairly good’, ‘fairly bad’* or *‘very bad’*. Further participants were asked how they would rate their sleep quality as a result of the coronavirus (COVID-19) situation during the past month. Again, the response categories were *‘very good’, ‘fairly good’, ‘fairly bad’* or *‘very bad’.*

### Other Risk or Protective Psychological Factors

#### Emotional Dysregulation

The Difficulties in Emotion Regulation Scale—Short Form (DERS-SF; Kaufman et al. 2016) was used to measure deficits in emotional regulation. The DERS-SF was developed from the original 36-item scale (DERS, Gratz and Roemer 2004). The DERS-SF contains 18 items rated on a 5-point Likert scale, ranging from 1 to 5. Items 1, 4 and 6 are reverse coded. The response categories were, ‘*almost never’* (1), ‘*sometimes’* (2), ‘*about half of the time’* (3), ‘*most of the time’* (4), and *‘almost always’* (5). The measure yields a total score as well as scores on six sub-scales. Each subscale reflects a different aspect of emotional dysregulation. These are (1) *‘non-acceptance’, (2) ‘difficulties with goal directed behaviour’, (3) ‘impulse control’, (4) ‘lack of emotional awareness’, (5) ‘lack of clarity’* and (6) *‘limited access to emotional regulation strategies’*. Higher scores indicate higher levels of dysregulation. In comparison to the original 36-item form, DERS-SF has been shown to have excellent psychometric properties, with internal reliability values for both the DERS-SF total scale and six subscales ranging from 0.78 to 0.91 in the original validation study (Kaufman et al. 2016). Additionally, Kaufman et al. (2016) indicated correlations between the DERS and DERS-SF ranged from 0.90 to 0.97 and indicated that the DERS and the DERS-SF shared 81–94% of their variance.

#### Loneliness

The UCLA Three-Item Loneliness Scale (Hughes et al. 2004) was used to measure subjective feelings of loneliness among the sample. The UCLA 3 item Loneliness Scale contains three questions derived from the full-scale UCLA Loneliness Scale (Version 3; Russell 1996). Each item measures one of three key dimensions of loneliness, (1) social connectedness, (2) relational connectedness and (3) self-perceived connectedness. The response categories are (1) *‘Hardly ever’*, (2) *‘Some of the time’* and (3) *‘Often’*. Higher scores across these items reflect higher levels of loneliness. The excellent psychometric properties of the both the long and short forms of the UCLA Loneliness scale are well documented (Hughes et al. 2004; Russell 1996). Additionally, participants were asked to indicate how often they felt lonely, using the same response categories as above. This was a bespoke question.

#### Social Support

The Perceived Social Support Questionnaire-Brief Form (Kliem et al. 2015) was used to assess participants perceived level of social support. The measure contains 6 items which are rated on a 5-point Likert scale ranging from 1 (*‘not true at all’*) to 5 (*‘very true’*). Higher scores reflect higher levels of perceived social support. Previous research supports the psychometric validity of the scale across a range of diverse populations (Kliem et al. 2015; Lin et al. 2019).

#### Meaning in Life

The Meaning in Life Questionnaire (MLQ; Steger et al. 2006) was used to measure the presence of and pursuit for meaning in life. The MLQ contains 10 items which correspond to one of two dimensions of meaning in life (1) ‘presence of meaning’ (which refers to the extent to which participants feel that their lives have meaning), and (2) ‘the pursuit of meaning’ (this refers to the extent to which participants try to find meaning and understanding in their lives; Steger et al. 2006). Each item is rated on a 7-point Likert scale ranging from 1 (*‘Absolutely True’*) to 7 (*‘Absolutely Untrue’*).

## Analytic Plan

Our analytic plan, for the current paper, was conducted across three complimentary phases as follows:

1. A series of descriptive analyses were conducted to present an overview of the key sociodemographic characteristics of the entire sample (*N* = 1989) for the baseline data of the COVID-19 Psychological Wellbeing Study.
2. A series of descriptive analyses were conducted to present an overview of the key sociodemographic characteristics by UK nation.
3. The sample distributions of key sociodemographic variables were examined in comparison to recent UK population distributions (gleaned from census data) by UK nation of residence; these were age, gender, ethnicity, economic activity, and household composition.

## Results

The COVID-19 Psychological Well-being Study was designed as a rapid data collection exercise across the UK population. This work was supported in part by funds from the School of Psychology at Queens University Belfast in Northern Ireland and the Department of Psychology at Glasgow Caledonian University, in Scotland. The recruitment target was 2000 participants in baseline. The total response rate was 2501 and after several exclusions were applied concerning data quality control (please see above methods section) the final effective sample size was 1989 participants.

Table 1 displays the key sociodemographic characteristics across both the overall sample (*N* = 1989) and by each nation. Both England and Wales were combined in order for Census comparison (see Table 2). Overall, the majority of the sample were female (*n* = 1392; 70.0%). Additionally, the majority of the sample were white (*n* = 1844; 92.7%). Further, the majority of the sample were employed full time (*n* = 879; 44.2%) and stated that they were not religious (*n* = 1143; 57.5%). Diversity was evident in relation to age, household income, marital status and educational attainment.

**Table 1:** Sociodemographic characteristics of the COVID-19 Psychological Wellbeing Study (reported by Total sample, England/Wales; Scotland & Northern Ireland)

		Total Sample (*N* = 1989)		England/Wales (*n* = 793)		Scotland (*n* = 726)		Northern Ireland (*n* = 470)	
		*N*	%	*n*	%	*n*	%	*n*	%
Gender	Female	1392	70.0	498	62.8	541	74.5	353	75.1
	Male	582	29.3	288	36.3	180	24.8	114	24.3
	Transgender	4	0.3	2	0.2	2	0.3	-	-
	Gender Variant	8	0.4	4	0.5	2	0.3	2	0.4
	Other	1	0.1	-	-	1	0.1	-	-
	Prefer not to say	2	0.1	1	0.1	-	-	1	0.2
Age group (years)	18–24	331	16.6	170	21.4	95	13.1	66	14.0
	25–34	659	33.1	261	32.9	231	31.8	167	35.5
	35–44	476	23.9	167	21.1	186	25.6	123	26.2
	45–54	291	14.6	112	14.1	106	14.6	73	15.5
	55–64	169	8.5	70	8.8	70	9.6	29	6.2
	65 and over	63	3.2	13	1.6	38	5.2	12	2.6
Household income	Less than average	746	37.5	347	43.8	264	36.5	135	28.8
	Average	900	45.2	352	44.4	315	43.6	233	49.8
	More than average	338	17.0	94	11.9	144	19.9	100	21.4
Ethnicity	White	1844	92.7	705	88.9	679	93.5	460	97.9
	Black/African/Caribbean	31	1.6	22	2.8	7	1.0	2	0.4
	Asian	59	3.0	40	5.0	14	1.9	5	1.1
	Mixed	40	2.0	19	2.4	20	2.8	1	0.2
	Other	9	0.5	3	0.4	5	0.1	1	0.2
	Prefer not to say	6	0.3	4	0.5	1	0.7	1	0.2
Religion	No Religion	1143	57.5	503	63.4	496	68.3	144	30.6
	Protestant	313	15.7	76	9.6	92	12.7	145	30.9
	Catholic	279	14.0	60	7.6	75	10.3	144	30.6
	Other Christian	142	7.1	94	11.9	30	4.1	18	3.8
	Buddhist	17	0.9	7	0.9	5	0.7	5	1.1
	Hindu	9	0.5	7	0.9	2	0.3	-	-
	Jewish	5	0.3	4	0.5	-	-	1	0.2
	Muslim	32	1.6	26	3.3	6	0.8	-	-
	Other	25	1.3	9	1.1	9	1.2	7	1.5
	Prefer not to say	24	1.2	7	0.9	11	1.5	6	1.3
Marital status	Single or never married	706	35.5	319	40.2	220	30.3	167	35.5
	Married or living with partner	1092	54.9	407	51.3	423	58.3	262	55.7
	Separated or divorced	101	5.1	42	5.3	37	5.1	22	4.7
	Widowed	25	1.3	6	0.8	16	2.2	3	0.6
	Other	54	2.7	13	1.6	26	3.6	15	3.2
	Prefer not to say	11	0.6	6	0.8	4	0.6	1	0.2
Highest level of education attained	No Qualifications	21	1.1	9	1.1	7	1.0	5	1.1
	Completed Secondary school to O-level/GCSE or similar	183	9.2	96	12.1	52	7.2	35	7.4
	Completed Secondary school to A-level or similar	319	16.0	180	22.7	72	9.9	67	14.3
	Certificate of Higher Education or similar (NVQ Level 4)	162	8.1	60	7.6	69	9.5	33	7.0
	Diploma of Higher Education or similar (NVQ Level 5)	126	6.3	35	4.4	61	8.4	30	6.4
	Undergraduate degree	647	32.5	275	34.7	235	32.4	137	29.1
	Postgraduate Degree	390	19.6	113	14.2	152	20.9	125	26.6
	Doctoral Degree	127	0.7	23	2.9	71	9.8	33	7.0
	Other	14	1.3	2	0.3	7	1.0	5	1.1
Employment^a^	Full-time	879	44.2	330	41.6	315	43.4	234	49.8
	Part-time	396	19.9	153	19.3	153	21.1	90	19.1
	Unemployed	173	8.7	96	12.1	51	7.0	26	5.5
	Self-Employed (full time or part time)	182	9.2	72	9.1	67	9.2	43	9.1
	Not able to work	64	3.2	31	3.9	20	2.8	13	2.8
	Retired	104	5.3	31	3.9	54	7.4	19	4.1
	Student	288	14.5	102	12.8	103	14.2	83	17.6

^a^ Individuals were able to endorse multiple responses for the employment question

**Table 2:** Comparing the socio-demographic characteristics of the COVID-19 Psychological Wellbeing Study baseline data (collected March/April 2020) to the socio-demographic characteristics of UK adult population (*N* = 1989)

		UK adult population (% distributions based on 2011 Census)			The COVID-19 Psychological Study Sample (%) (± % difference between survey sample and population)		
		England/Wales (N = 44,105,900)	Scotland (N = 4,252,806)	NI (N = 1,380,100)	England/Wales (N = 793)	Scotland (N = 726)	NI (N = 470)
Country of residence^a^		88.7%	8.6%	2.8%	39.9% (− 48.8%)	36.5% (+ 27.9%)	23.6% (+ 20.8%)
Gender^a,b^	Female	51.4%	52.8%	51.7%	62.8% (+ 11.4%)	74.5% (+ 22.2%)	75.1% (+ 23.4%)
	Male	48.6%	47.8%	48.3%	36.8% (− 11.8%)	24.8% (− 23.0%)	24.3% (− 24.0%)
Ethnicity^a^	White	87.8%	96.4%	98.5%	88.9% (+ 1.1%)	93.5% (− 2.9%)	97.9% (− 0.6%)
	Asian	6.9%	2.5%	1.0%	5.0% (− 1.9%)	1.9% (− 0.6%)	1.1% (+ 0.1%)
	Black/African/Afro-Caribbean	2.9%	0.6%	0.2%	2.8% (− 0.1%)	1.0% (+ 0.4%)	0.4% (+ 0.2%)
	Mixed	1.4%	0.2%	0.2%	2.4% (+ 1.0%)	2.8% (+ 2.6%)	0.2% ( =)
	Other	0.9%	0.2%	0.1%	0.4% (− 0.5%)	0.7% (+ 0.5%)	0.2% (+ 0.1%)
Economic activity^c^	Employed full-time	36.0%	37.5%	33.9%	41.6% (+ 5.6%)	43.4% (+ 5.9%)	49.8% (+ 15.9%)
	Employed part-time	12.9%	14.8%	12.5%	19.3% (+ 6.4%)	21.1% (+ 6.3%)	19.1% (+ 6.6%)
	Self Employed (Full- or part-time)	9.2%	7.2%	9.4%	9.1% (− 0.1%)	9.2% (+ 2.0%)	9.1% (− 0.3%)
	Unemployed	4.1%	5.2%	4.7%	12.1% (+ 8.0%)	7.0% (+ 1.8%)	5.5% (+ 0.8%)
	Retired	22.0%	22.9%	12.2%	3.9% (− 18.1%)	7.4% (− 15.5%)	4.1% (− 8.1%)
	Student	8.6%	5.2%	9.4%	30.5% (+ 21.9%)	14.2% (+ 9.0%)	17.6% (+ 8.2%)
	Unable to work	4.0%	4.9%	6.9%	3.9% (− 0.1%)	2.8% (− 2.1%)	2.8% (− 4.1%)
	Other	6.3%	5.3%	7.0%	3.3% (− 3.0%)	5.5% (+ 0.2%)	4.0% (− 3.0%)
Household composition^d^	Adult only	60.0%	62.2%	-	62.3% (+ 2.3%)	65.0% (+ 2.8%)	60.7% (NA)
	Other	40.0%	37.8%	-	37.7% (− 2.3%)	35.0% (− 2.8%)	39.2% (NA)
Education^e^	No qualification	23.4%	27.6%	30.2%	1.1% (− 22.3%)	1.0% (− 26.6%)	1.1% (− 29.1%)
	GCSE (Level 1 & 2)	29.4%	23.8%	27.4%	12.1% (− 17.3%)	7.2% (− 16.6%)	7.4% (− 20.0%)
	A-Level (Level 3)	35.4%	14.8%	12.8	22.7% (− 12.7%)	9.9% (− 4.9%)	14.3% (+ 1.5%)
	Level 4 and above	31.8%	26.9%	24.5%	63.8% (+ 32.0%)	81.0% (+ 54.1%)	76.2% (+ 51.7%)
	Other	5.8%	-	8.8%	0.2% (− 5.6%)	1.0% (NA)	1.1% (− 7.7%)
Age^a^	18–24	11.9%	11.9%	12.8%	21.4% (+ 9.5%)	13.1% (+ 1.2%)	14.0% (+ 1.2%)
	25–34	17.1%	15.7%	17.7%	32.9% (+ 15.8%)	31.8% (+ 16.1%)	35.5% (+ 17.8%)
	35–44	17.8%	17.3%	18.4%	21.1% (+ 3.3%)	25.6% (+ 8.3%)	26.2% (+ 7.8%)
	45–54	17.5%	18.5%	18.0%	14.1% (− 3.4%)	14.6% (− 3.9%)	15.5% (− 2.5%)
	55–64	14.9%	15.7%	14.0%	8.8% (− 6.1%)	9.6% (− 6.1%)	6.2% (− 7.8%)
	65 +	20.9%	20.9%	19.1%	1.6% (− 19.3%)	5.2% (− 15.7%)	2.6% (− 16.5%)

Note: All figures gleaned from the census data have been confirmed by the authors. Nation data presented refers ONLY to the COVID-19 Psychological Wellbeing Study ^a^ Source: 2011 Census population estimates for adults aged 18 + for England/Wales, Scotland and Northern Ireland ^b^ Note: 0.8% of individuals in the current survey stated another gender (i.e. transgender, gender variant, prefer not to say, other) and are therefore not included in this comparison. Please not participants were given to option to state they did not want to disclose their gender, these were excluded from the above table ^c^ Source: 2011 Census population estimates for adults aged 16 + for England/Wales and Scotland; Northern Ireland age 16–74 years (no other breakdown of age publicly available). Note: in current survey respondents could endorse multiple response options ^d^ Source: 2011 Census population estimates for adults aged 25 + years for England/Wales and Scotland; Northern Ireland provides publicly available data on household composition for the household reference person only (N = 703,275), not for all adults aged 18 + years, and therefore a comparison to survey for household composition is not feasible ^e^ Education: Source 2011 Census population estimates for adults aged 16 + for England/Wales, Scotland and Northern Ireland

In order to assess the representativeness of the COVID-19 Psychological Wellbeing Study sample to the UK general population, it was compared to data from the UK Census 2011 for adults aged 18 years + . In some cases, where estimates for those 18 and older were not available, alternative comparisons were made (e.g. 16 + or 25 + years). These are noted in the table footnotes section. Specifically, the sample was compared by UK country of residence, gender, ethnicity, economic activity, household composition, age and education level. Although this method is inexact due to changes within the population in the past decade, the 2011 Census contains publicly available information on all sociodemographic variables of interest. These results are presented in Table 2, modelled on that of McBride et al. (2020; preprint). Results are presented across the UK nations, however, in line with the Census statistics, information on England and Wales are presented together.

In brief, the COVID-19 Psychological Wellbeing Study sample was not representative of the UK population as a whole (by country of residence) or within the UK nations (by sociodemographic characteristics). Respondents from Scotland and NI were oversampled in the study while those from England/Wales were underrepresented. Within each nation, females were oversampled and males under-sampled, particularly those from Scotland and NI. Ethnicity comparisons revealed that this was the variable which most closely represented the ethnic profiles across the UK nations. Economic activity was difficult to compare to the Census statistics due to the survey methodology. Within the survey, respondents were able to endorse multiple options related to their employment and studying. As such, respondents could report being employed and a student, or being employed part-time and being self-employed. However, a crude comparison to the Census data suggests that employed individuals and students were oversampled, while those who are unable to work or are retired were undersampled. This was similarly reflected in age group comparisons; younger individuals (particularly those aged 25 – 34) made up a greater proportion of the sample than expected from the population estimates, while there was a substantial deficit in the number of older adults (particularly those aged 65 years +) within the sample. As noted by McBride et al. (2020; preprint), an accurate comparison of household composition was not achievable from the NI Census data. However, the household composition of England/Wales and Scotland was similar to the Census estimates.

Table 3 displays summary statistics regarding the housing conditions and composition of the COVID-19 Psychological Wellbeing Study respondents (*N* = 1989). Overall, the majority of the sample lived in a city (*n* = 683; 34.4%). Regarding housing conditions, the majority of the sample lived in a house (*n* = 1476; 74.2%), owned their place of residence with a mortgage (*n* = 732; 36.9%) or rented their place of residence (*n* = 674; 34.%). In terms of housing composition, majority of respondents stated at least two adults lived in their home (including them; 72.6%). Further, most of the sample did not have any children under the age of 18 in their place of residence (*n* = 1248; 62.9%). Finally, almost half of the overall sample did not own a pet (*n* = 919; 46.2%), with diversity evident across those who did own pets.

**Table 3:** Housing conditions and composition of the COVID-19 Psychological Wellbeing Study respondents (reported by full sample and by England/Wales; Scotland & Northern Ireland)

Housing conditions and composition		Total (*N* = 1989)		England / Wales (*n* = 793)		Scotland (*n* = 723)^a^		Northern Ireland (*n* = 470)	
		*N*	%	*N*	%	*N*	%	*n*	%
Housing area	Isolated dwelling	65	3.3	9	1.1	12	1.7	44	9.4
	Hamlet	33	1.7	6	0.8	12	1.7	15	3.2
	Village	336	16.9	140	17.7	133	18.4	63	13.5
	Small town	517	26.1	245	30.9	175	24.2	97	20.7
	Large Town	350	17.6	159	20.1	111	15.4	80	17.1
	City	683	34.4	234	29.5	280	38.7	169	36.1
Type of dwelling	House	1476	74.2	628	79.2	433	59.9	415	88.7
	Room(s) in shared house (e.g., lodger)	41	2.1	19	2.4	11	1.5	11	2.4
	An apartment or flat in a block	436	22.0	132	16.6	269	37.2	35	7.5
	Student Halls	12	0.6	4	0.5	5	0.7	3	0.6
	Residential Home	5	0.3	2	0.3	1	0.1	2	0.4
	Other	14	0.7	8	1.0	4	0.6	2	0.4
Housing tenure	Owned (outright)	325	16.4	138	17.4	119	16.5	68	14.5
	Owned (with a mortgage)	732	36.9	251	31.7	302	41.8	179	38.2
	Shared ownership (part rent/owned/mortgage)	30	1.5	10	1.3	9	1.2	11	2.4
	Rented	674	34.0	279	35.2	247	34.2	148	31.6
	Living rent free (e.g. with family)	187	9.4	100	12.6	39	5.4	48	10.3
	Other	36	1.8	15	1.9	7	1.0	14	3.0
No. of adults living in the household (18 yrs +)	1	399	20.1	142	17.9	167	23.1	90	19.2
	2	1041	72.6	382	48.2	409	56.6	250	53.4
	3	325	89.0	156	19.7	97	13.4	72	15.4
	4	152	7.7	73	9.2	40	5.5	39	8.3
	5 +	67	3.4	40	5.0	10	1.4	17	3.6
No. of Children living in the household (> 18yrs)	0	1248	62.9	494	62.3	470	65	284	60.7
	1	347	17.5	158	19.9	120	16.6	69	14.7
	2	290	14.6	111	14.0	104	14.4	75	16.0
	3	79	4.0	27	3.4	23	3.2	29	6.2
	4 +	20	1.0	3	0.4	6	0.8	11	2.3
No. of bedrooms^b^	1	168	8.5	66	8.3	84	11.6	18	3.8
	2	522	26.3	209	26.4	242	33.5	71	15.2
	3	787	39.7	311	39.2	260	36.0	216	46.2
	4	392	19.8	155	19.5	113	15.6	124	26.5
	5 +	115	5.8	52	6.6	24	3.3	39	8.3
Pets living in your house^c^	Dog	618	31.1	242	30.5	196	27.0	180	38.3
	Cat	515	25.9	229	28.9	164	22.6	122	26.0
	Bird	34	1.7	20	2.5	6	0.8	8	1.7
	Fish	119	6.0	61	7.7	31	4.3	27	5.7
	Other	144	7.2	58	7.3	56	7.7	30	6.4
	Does not own a pet	919	46.2	359	45.3	368	50.7	202	43.0

Note: ^a^ n = 3 participants in the Scottish sample had missing data across these variables and were therefore excluded; ^b^ n = 2 participants in the N.I sample had missing data across these variables and were therefore excluded;^c^ Participants could choose more than one pet and n = 5 participants has missing data across the entire sample for this question

Table 4 displays summary statistics regarding keyworker classification for the entire sample (*N* = 1989) and by each nation. Overall, 37.4% (*n* = 746) of respondents stated they were employed within one of the government assigned key worker roles at the time of survey completion. Of these 746 respondents, majority were keyworkers in the area of health and social care (*n* = 222), followed closely by education/childcare (*n* = 179). This was consistent across each of the nations.

**Table 4:** Keyworker status reported by respondents across nations

Keyworker classification	Total sample (*N* = 1989)		England / Wales (*n* = 793)		Scotland (*n* = 725 ^a^)		Northern Ireland (*n* = 470^b^)	
	*n*	%	*n*	%	*n*	%	n	%
Health and social care	222	11.2	68	8.6	85	11.7	69	14.7
Education and childcare (e.g. childcare, support and teaching staff, social workers and those specialist education professionals)	179	9.0	75	9.5	55	7.6	49	10.5
Transport (e.g. air, water, road and rail passenger and freight transport modes)	24	1.2	8	1.0	12	1.7	4	0.9
Key public services (e.g. the justice system, charities and workers delivering key frontline services)	62	3.1	17	2.1	22	3.0	23	4.9
Local and national government	64	3.2	22	2.8	28	3.9	14	3.0
Food and other necessity goods (e.g. food production, processing, distribution, sale and delivery)	87	4.4	33	4.2	28	3.9	26	5.6
Public safety (e.g. armed forces personnel, fire and rescue service employees, police)	23	1.2	8	1.0	7	1.0	8	1.7
Utilities, communication and financial services (e.g. workers in banks, building societies and financial market infrastructure), the oil, gas, electricity and water sectors, the information technology and data infrastructure sector)	82	4.1	34	4.3	34	4.7	14	3.0
None of these – I am not a keyworker	1243	62.6	528	66.6	454	62.6	261	55.8

^a^ n = 1 participant within the Scottish data had missing data and therefore was excluded from the data presented; ^b^ n = 2 participants form the N.I data has missing data and were therefore excluded from the data presented

## Discussion

The purpose of the current study was to provide a technical overview of the design and procedures involved in initiating the COVID-19 Psychological Wellbeing Study. Furthermore, details of the measures utilised in the baseline survey and the sociodemographic characteristics of the sample are presented. Given the expected widespread impact of the pandemic and its associated impact on mental health, the survey was implemented rapidly to allow for a comprehensive assessment of changes in mental health as situation unfolded within the UK.

### Alignment with Mental Health Research Priorities

As previously mentioned, the current study was designed around key research priorities as identified from previous epidemic and pandemic research (e.g. SARS), the broader literature surrounding the impact of traumatic events and consideration of topical issues that were of public interest. Moreover, the focus of the COVID-19 Psychological Wellbeing Study aligns well with a recent report published in The Lancet Psychiatry (Holmes et al. 2020). Although this position paper on mental health priorities during the COVID-19 pandemic was published after the current study had been launched, many of the key areas identified by the authors are covered. Holmes et al. (2020) identified a number of immediate and long-term mental health research priorities both at an individual and a population level. The nature of the current study will allow for opportunities to focus on some of these priorities, namely, monitoring and reporting of common mental health problems, identifying groups who are particularly vulnerable to psychological distress at this time, determining the mechanism which underlie these mental health problems (i.e. risk and protective factors), ascertaining the longer term consequences of the pandemic across the population and within vulnerable groups, and investigating the effect of repeated pandemic-related media consumption on mental health. Such research can inform the design and development of a range of appropriate digital interventions both a population level and bespoke interventions for specific groups of individuals.

Mental health services have been highlighted as an essential part of governments’ responses to COVID-19 (United Nations 2020). The UK Government has published a recovery strategy for COVID-19 (UK Government 2020) which acknowledges the potential impact of these recent societal changes on the nation’s mental health. Although the strategy promises improvements to, and funding for, health and social care settings in order to facilitate safer access to services in future (e.g. delivering service digitally), at the time of writing, no specific UK mental health strategy for COVID-19 has been put in place. A range of mental health campaigns have been launched, however, for example, *Every Mind Matters* (Public Health England 2020), *How are you doing?* (Public Health Wales 2020), *Clear Your Head* (Scottish Government 2020), while the Department of Health in NI launched a Mental Health Action Plan (Department of Health 2020) in response to COVID-19 on 19 May. It is clear that a focused mental health strategy will be needed in light of the current pandemic, either UK wide or across nations. Longitudinal research, such as that of the current study, may help inform these strategies and campaigns by highlighting key areas of attention or concern and specific groups who are experiencing the most distress.

### Strengths

The current study has many strengths, particularly in respect to study design. Firstly, a large range of variables (sociodemographic, psychological, health, COVID-19 specific, etc.) were covered in the initial baseline survey and follow-up surveys. This will yield a vast and diverse amount of information which can be used to help garner a better, more comprehensive understanding of the impact of these unique circumstances longitudinally. Indeed, the topics covered within the survey were empirically derived, based on previous epidemic and pandemic research and matched many of the research priorities previously mentioned. For example, Holmes et al. (2020) identified at least eight groups of individuals who may be particularly vulnerable to experiencing mental distress at this time, such as front line workers, people on low income or those with financial insecurities, children and young people, etc. Therefore, the large range of sociodemographic topics covered will allow for a wide-ranging investigation of at-risk groups. Additionally, psychometrically valid and frequently used measures of anxiety, depression and PTSD were included in the study which may aid future comparisons.

Furthermore, although no specific COVID-19 measures were available at the time of survey design, the COVID-19 specific items were based on reliable sources of COVID-19 information (e.g. WHO) and were reviewed by several academics before being included in the study. Moreover, the research team acted rapidly as the COVID-19 context evolved to ensure topics which had not been included within the baseline survey, but subsequently became areas of interest or research priorities (e.g. attitudes towards vaccines), were included within the follow-up surveys. The multi-phase survey design aimed to facilitate a comprehensive data collection strategy and was an additional strength to the study. Data were collected intensively from survey launch; all respondents completed the baseline while approximately half completed weekly follow-up surveys for the next month. This was followed by three anticipated monthly follow-up surveys for the full sample. This strategy allows for a comprehensive overview of mental health and wellbeing for the first month of the lockdown period in which most people’s daily lives had changed dramatically, and also for an investigation of the more nuanced findings over time and as restrictions eased in the following months.

Finally, as openly acknowledged throughout, this study did not aim to collect representative UK sample. However, efforts were made post-data collection to assess the degree of representativeness within and across the UK nations. Although ultimately the data was not representative, a substantial number of responses were gathered from England, Scotland and NI, while few came from Wales. As such, a more diverse UK sample was collected in relation to country of residence and at the time of writing, this is the largest known data collection exercise on COVID-19 and mental wellbeing in NI.

### Limitations

A number of limitations are important to consider in the context of the current study. Arguably, the main limitation of the study is that the data is not representative of the UK population as a whole, in terms of country of residence, or of the individual UK nations in terms of their sociodemographic characteristics. Therefore, the findings may not be generalisable to the wider UK population as a whole. However, as discussed above, there were certain strengths unique to the aims of the study by oversampling those from Northern Ireland and Scotland. It is also important to note that the data pertaining to the current study is modest in nature as compared to some ongoing data collection efforts in the UK (Fancourt et al. 2020), but similar in sample size of others (McBride et al. 2020). The aim of the COVID-19 Psychological Wellbeing study was to collect data on 2000 participants. This decision was based on the fact that the research was being conducted in the absence of external funding and the research team chose not to apply for external funding.

Additionally, while the utilisation of online survey methods was deemed the only safe way to gather such data on a large scale amidst a pandemic and has the added benefit of increasing accessibility to those groups who would be difficult to access via other means (Wright 2005), it is also important to mention that our sampling strategy may result in self-selection bias (Bethlehem 2010). All participants involved in the current study were recruited via a social media campaign or via Prolific panel data, each of these options requires the participant (1) to opt in first of all and (2) have access to an internet connection and equipment to take the survey online. Therefore, specific groups may be underrepresented because they do not have an internet connection, computer/smartphone device, social media profile or simply do not wish to take part in the research (Bethlehem 2010).

Given the unprecedented nature of this pandemic, it is important to also mention a number of important risk factors and experiences that were not queried in the baseline survey. We did not examine general physical health and exercise, abuse or maltreatment within the home, interpersonal violence, more in depth exploration of specific disorders such as OCD, health anxiety or diabetes nor peoples experiences of medical care if required for a COVID19 diagnosis. Of note, we did query ‘how worried people were about the ability of the health system to care for coronavirus (COVID-19) patients if the situation worsens’. A large proportion of respondents were ‘extremely’ worried about this (*n* = 720; 36.5%) or quite a bit worried (n = 641, 32.5%).

Given the nature of the pandemic, the researchers were responsive to COVID-19 topics of public and scientific interest and inserted additional topics into future waves of data collection; for example attitudes to a COVID-19 vaccine (if one was to become available) and problematic drinking behaviours.

Regarding the questions in the survey itself, it is possible that participants, when asked about *‘self-isolation’*, may have had different interpretations regarding the specific meaning, either due to their own personal understanding or the rapidly changing government guidance as the situation progressed.

Finally, all measures used in relation to the current study were self-report, therefore it cannot be ruled out that the respondents may have been influenced by their willingness or indeed unwillingness to report correctly, which therefore may potentially bias the results (Weiss et al. 2018).

### Ongoing Data Collection Efforts

Since the implementation of the baseline survey on the March 23^rd^ substantial progress has been made. Regarding the respondents who were recruited via the social media campaign, a further 4 follow up surveys have been completed (1 week, 2 week, 3 week and 1 month post baseline completion). All respondents recruited via Prolific have also now completed a 1 month and 2 month follow up survey.

### Future Research Directions

The circumstances surrounding this pandemic are rapidly changing and individuals are constantly adapting to change and challenges in their lives and routines: distancing from loved ones, working from home, job losses, and at times inability to grieve in the usual way. It is likely that the lasting effects of this pandemic may not become apparent until months down the line, or they may fluctuate in peaks and troughs in relation to key events. For example, a spike in poor mental health following lockdown and an ease in COVID-19 concerns and worries with time (C19PRC 2020; Fancourt et al. 2020). It is therefore imperative that longitudinal and prospective research is prioritised in order to map these changes across the UK. In order to address this, as aforementioned, the COVID-19 Psychological Wellbeing Study aims to conduct both weekly and monthly follow-up surveys in order to provide a thorough investigation of how the mental health and wellbeing of individuals has been impacted by (1) the outbreak itself, (2) the first month of the lockdown period and (3) the period following the ease of lockdown restrictions, allowing for more nuanced study.

Additionally, it is essential such research aims to embrace and investigate the complexity of studying mental health and wellbeing during these unprecedented times. Therefore, not only studying causal links but also the mechanisms that influence the relationship between certain risk factors and mental health problems, e.g., social isolation and loneliness, emotional regulation, coping strategies, certain demographic risks such as living alone, financial and employment concerns etc. Furthermore, certain groups of individuals may need specific examination (e.g. parents, key workers, those who are shielding etc.).

Future research should strive to collect data allowing cross-nation comparisons within the UK. This will be of interest given that various Government bodies have taken different approaches within the four nations and there are varying pre-covid social, economic and cultural differences across the UK nations; some of which have the potential to impact on population wellbeing. Moreover, this extends to cross-country comparisons given countries have varying approaches to lockdown restrictions and the ease of these restrictions. The COVID-19 Psychological Wellbeing Study affords the opportunity to make viable cross-country comparisons with partners in the US, Israel and Australia because of collaboration on and direct sharing of the study protocol and measures with in-country investigators who are undertaking their own data collection efforts.

Given these unprecedented times, qualitative research exploring the unique lived experiences of particular vulnerable/at risk groups such as those with pre-COVID19 mental health concerns or those working on the front line is essential. The COVID-19 Psychological Wellbeing Study has acknowledged this need and has recently launched a sister qualitative study – *‘The Caring for the COVID-19 Carers, Key Workers, and their families Study’.* This study aims to understand the perceptions and experiences of both healthcare professionals and their family members during the outbreak of COVID-19. At present, the data collection is nearing completion and a separate paper will be published in due course outlining the methodology and research findings regarding this.

## Conclusion

In sum, the COVID-19 Psychological Wellbeing Study aims to rapidly assess and monitor the psychosocial impact of the COVID-19 pandemic on UK residents. At the time of writing, this is the largest known data collection exercise on COVID-19 and mental wellbeing in NI. Given the focus on both a quantitative longitudinal multi wave design, and a sister qualitative study we believe this programme of research is in a unique position to make a significant contribution to the growing body of literature to help understand the psychological impact of this pandemic.

## Data Availability

The participants did not give consent for their data to be made publicly available. Derived data supporting the findings of this study will be made available from the corresponding author on reasonable request.

## Footnotes

1 Please note that these figures refer to deaths of people who had a confirmed positive COVID19 test result. The actual number of deaths as a result of COVID19 are likely much larger; the official figures, cited here, do not include individuals who had not been tested at the time of their death, those who tested positive from a non-NHS or Public Health laboratory, those who initially tested negative but subsequently caught the virus and died without a subsequent positive test and, as an indirect result of the virus, as seen in the large numbers of excess deaths during this time. Furthermore, it is also important to note that small differences in the numbers of deaths recorded are present depending on where and when the data were sourced. There can be delays in a death occurring and it being reported in the official statistics for a number of reasons (e.g. awaiting test results). Thus, the figures presented here are those that the Department of Health and Social Care had recorded by that date. Subsequently these figures were revised to include additional deaths that had taken place by that date but had not been officially recorded as Covid19 deaths. https://coronavirus.data.gov.uk/about
